# Unintentional exposure to terrestrial pesticides drives widespread and predictable evolution of resistance in freshwater crustaceans

**DOI:** 10.1111/eva.12584

**Published:** 2018-01-20

**Authors:** Kaley M. Major, Donald P. Weston, Michael J. Lydy, Gary A. Wellborn, Helen C. Poynton

**Affiliations:** ^1^ School for the Environment University of Massachusetts Boston Boston MA USA; ^2^ Department of Integrative Biology University of California Berkeley CA USA; ^3^ Center for Fisheries, Aquaculture and Aquatic Sciences Carbondale IL USA; ^4^ Department of Zoology Southern Illinois University Carbondale IL USA; ^5^ Department of Biology University of Oklahoma Norman OK USA

**Keywords:** evolutionary ecotoxicology, *Hyalella azteca*, insecticide resistance, nontarget, parallel evolution

## Abstract

Pesticide runoff from terrestrial environments into waterways is often lethal to freshwater organisms, but exposure may also drive evolution of pesticide resistance. We analyzed the degree of resistance and molecular genetic changes underlying resistance in *Hyalella azteca*, a species complex of freshwater crustaceans inadvertently exposed to pesticide pollution via runoff. We surveyed 16 waterways encompassing most major watersheds throughout California and found that land use patterns are predictive of both pyrethroid presence in aquatic sediments and pyrethroid resistance in *H. azteca*. Nonsynonymous amino acid substitutions in the voltage‐gated sodium channel including the M918L, L925I, or L925V confer resistance in *H. azteca*. The most frequently identified mutation, L925I, appears to be preferred within the species complex. The L925V substitution has been associated with pyrethroid resistance in another insect, but is novel in *H. azteca*. We documented a variety of pyrethroid resistance mutations across several species groups within this complex, indicating that pyrethroid resistance has independently arisen in *H. azteca* at least six separate times. Further, the high frequency of resistance alleles indicates that pesticide‐mediated selection on *H. azteca* populations in waterways equals or exceeds that of targeted terrestrial pests. Widespread resistance throughout California suggests current practices to mitigate off‐site movement of pyrethroids are inadequate to protect aquatic life from negative ecological impacts and implies the likelihood of similar findings globally.

## 1 INTRODUCTION

Widespread, global pesticide use has had devastating effects on ecosystems through impacts to keystone species such as pollinators, alterations in food webs, endocrine disruption in vertebrates, and indirect impacts to ecosystem function (Kohler & Triebskorn, 2013). Many target pest species have evolved resistance to pesticides, thereby evading their effects (Dong et al., 2014; Ffrench‐Constant, 2013; Ffrench‐Constant, Daborn, & Le Goff, 2004). Some nonpest species that encounter pesticides only incidentally may also evolve resistance, but the role of evolutionary processes in rescuing and restoring these nontarget populations is not well understood (Kohler & Triebskorn, 2013). Even in species with large populations and the ability to respond rapidly through adaptation, fitness costs imposed by the adaptations themselves may reduce the population's ability to respond to other stressors (Whitehead, Clark, Reid, Hahn, & Nacci, 2017) undoubtedly present in the highly modified ecosystems these species inhabit (Klerks & Levinton, 1989b; Reid et al., 2016; Whitehead et al., 2017). In addition, pollution tolerance conferred via adaptation (Amiard‐Triquet, Rainbow, & Romeo, 2011) has the potential to alter wild populations through genomewide changes to genetic diversity or changes in allele frequencies (Bickham, 2011). The field evolutionary toxicology has emerged to better understand these and other effects of anthropogenic contaminants on the genetics of natural populations (Bickham, 2011; Bickham & Smolen, 1994), although documented cases of adaptation to pollution are relatively few. However, some foundational examples of evolution to pollution in natural populations include evolved metal tolerance in oligochaete worms from the Foundry Cove in New York (Klerks & Levinton, 1989a, 1989b) and the polychlorinated biphenyl (PCB) and dioxin‐like chemical tolerance in killifish (Reid et al., 2016; Whitehead et al., 2017) and Atlantic tomcod (Wirgin et al., 2011).

Until recently, little evidence existed to implicate pesticides as strong selective factors capable of driving evolutionary change in nontarget wild populations (Kohler & Triebskorn, 2013). However, in the Central Valley of California, we documented levels of pyrethroid pesticide resistance of up to 550‐fold in some populations of epibenthic amphipods within the *Hyalella azteca* species complex (Weston et al., 2013). In addition to a pyrethroid‐resistant phenotype, resistant populations also harbored one of two mutations in the voltage‐gated sodium channel (Vgsc: the target site for pyrethroids) previously identified in pyrethroid‐resistant pest insects: methionine‐to‐leucine at position 918 (M918L) or leucine‐to‐isoleucine at position 925 (L925I; Weston et al., 2013). North American amphipods classified as *H. azteca* are now widely recognized to consist of a highly diverse, cryptic species complex (Witt & Hebert, 2000; Witt, Threloff, & Hebert, 2006) that can be differentiated from one another by genetic sequencing. Given that *H. azteca* (*sensu lato*) is a diverse species complex, it is especially interesting to note that these mutations have occurred independently and repeatedly in multiple species within this species complex (Weston et al., 2013). Parallel evolution of resistance mutations among insects and pyrethroid‐resistant *H. azteca* suggests similar selective pressures experienced in both the targeted pest and the nontarget, ecologically important amphipod unintentionally exposed to pesticides via runoff. In fact, waterways in urban, residential, and agricultural areas of California often have water or sediment concentrations of pyrethroids that exceed toxicity thresholds for sensitive, wild‐type *H. azteca* (Amweg, Weston, You, & Lydy, 2006; Holmes et al., 2008; Phillips et al., 2012; Weston, Holmes, & Lydy, 2009; Weston, Holmes, You, & Lydy, 2005; Weston & Lydy, 2012). Taken together, these studies indicate that pesticides entering waterways via runoff have sufficient toxicity to alter the evolutionary trajectory of arthropod populations by strongly selecting for resistance mutations.

A long‐standing question in molecular evolution is to what extent is evolutionary change predictable. Examples of parallel evolution, especially prevalent in the insecticide resistance literature (Ffrench‐Constant et al., 2004), support the hypothesis of predictable molecular evolution (Stern, 2013). For example, the *vgsc* is a common target for pyrethroid resistance, with over 120 documented cases of resistance developing across arthropods, mostly in pest species (Dong et al., 2014). In addition, a primary role of pesticide management practices is to minimize what is considered the inevitable development of resistance (Feyereisen, Dermauw, & Van Leeuwen, 2015; Palumbi, 2001). Therefore, insecticide resistance provides an interesting study system to explore the predictability of evolution in two dimensions: where evolution is likely to occur, and what molecular solution is likely to be utilized. Further, studying insecticide resistance in *H. azteca* from California is an ideal system for studying parallel evolution as a result of anthropogenic pollution. Because of required documentation of agricultural pesticide use in California (CDPR, 2015), the relationship between pyrethroid exposure and evolved resistance in wild populations of *H. azteca* can be established more easily than for other chemicals for which use is not recorded. In the wild, species classified as *H. azteca* are the most abundant amphipod found across North American (Batzer, Rader, & Wissinger, 1999), and as obligate aquatic macroinvertebrates, they have low dispersal compared to many aquatic insects (Stutz, Shiozawa, & Evans, 2010). Such low dispersal lends itself to studying populations exposed to localized pollution, and ensures that gene flow among populations remains low. Further, *H. azteca* exists as a species complex. Because there is sufficient species‐level genetic diversity among some pyrethroid‐resistant lineages, we can be certain that independent, parallel evolutionary events confer resistance phenotypes (Weston et al., 2013).

But just how common and predictable is pesticide‐driven evolution in nontarget organisms? Pyrethroids are used worldwide in agricultural, residential, and landscaping settings, leading to potential exposure of wild *H. azteca* throughout North America, and other amphipods globally. Without systematic screening of populations, it is not possible to determine the prevalence of these adaptive responses, nor ultimately understand the ecological implications associated with the strong selective pressure imposed by an anthropogenic pollutant. In the current study, we developed a genotyping assay that can be widely used within the *H. azteca* species complex to link pyrethroid resistance phenotypes to resistance alleles at the M918 and the L925 voltage‐gated sodium channel loci. We then investigated the widespread geographic distribution of pyrethroid resistance in populations of *H. azteca* throughout California to determine whether expected pyrethroid use on terrestrial land is predictive of pyrethroid resistance.

## 2 MATERIALS AND METHODS

### 2.1 Collection of *H. azteca*

Collection sites were selected using the California Environmental Data Exchange Network (CEDEN, 2015) to identify locations with high abundances of *H. azteca* sp., with a few additional sites selected based on prior work, or reconnaissance of water bodies in regions of interest. Based largely on the land use of nearby areas, we a priori classified eight sites as “low pyrethroid use (LowPU) expected” and eight sites as “high pyrethroid use (HighPU) expected” (see Supplemental Methods for classification details; Table S1) with the final site list providing a broad distribution of locations throughout California. In selecting sites, preference was given to sites having no direct hydrological connection between them through which *H. azteca* could migrate, so that opportunities for gene flow between sites would be very limited. Each site was in a separate watershed, either flowing to the Pacific Ocean, the salinity of which *H. azteca* cannot tolerate, or in an endorheic basin with no outlet (see Supplemental Methods for a few exceptions). All collections occurred between October 2014 and August 2015. *H. azteca* were collected using a D‐framed net and transported with aeration to UC Berkeley for toxicity testing and preservation for genetic analysis. Preservation in concentrated ethanol was done on site if the number of individuals collected were not sufficient for toxicity testing. Surficial sediment (0–2 cm) was also collected at each site for pesticide analysis, selecting the finest‐grained material available.

### 2.2 Pesticide sediment analyses

Sediment samples collected from the field sites were sent to Southern Illinois University for pesticide analyses. These analyses followed methods previously developed in our laboratory and detailed elsewhere (Weston, Chen, & Lydy, 2015; You, Weston, & Lydy, 2008). Target pesticides for sediments included the pyrethroid insecticides bifenthrin, cyfluthrin, cyhalothrin, cypermethrin, deltamethrin, esfenvalerate, permethrin, and tefluthrin. Prior to extraction, frozen sediment was freeze‐dried (Labconco Corporation, Kansas, MO) at −80°C for 24 hr. Approximately 5 g of dry sediment was mixed with 1 g of silica and 2 g of copper powder, and surrogate standards (4,4′‐dibromooctafluorobiphenyl (DBOFB) and decachlorobiphenyl (DCBP)) were added at this time. Sediment samples were extracted using a matrix‐dispersive accelerated solvent extraction method (You et al., 2008; see Supplemental Methods).

Confirmation of nominal water concentrations for cyfluthrin for the laboratory toxicity tests was performed so that all concentration data could be reported based on actual concentrations. Briefly, surrogates DBOFB and DCBP were added to the samples, and water was liquid:liquid extracted three times with 60 ml dichloromethane, with one 60 ml aliquot also used to rinse the sample bottle to remove any target insecticide that may have partitioned to the glass. The combined extracts were concentrated to 1 ml in hexane and analyzed following Wang, Weston, & Lydy (2009). The extract cleanup for the water samples was the same as used for the sediment extracts.

Final extracts were analyzed using an Agilent 6850 gas chromatograph 5975 XL mass spectrometer (GC‐MS; Agilent Technologies, Palo Alto, CA) with negative‐ion chemical ionization and selected‐ion monitoring. Inlet, ion source, and quadrupole temperatures were 260, 150, and 150°C, respectively. An HP‐5 MS column (30 m × 0.25 mm × 0.25 μm film thickness) was used for separation of the analytes using helium as a carrier gas with the flow rate set at 1.8 ml/min (see Supplemental Methods for additional details). Quality assurance for the field‐collected samples included a blank, laboratory control spike, matrix spike, matrix spike duplicate, and field duplicate, all run with every batch of 20 samples.

Sediment organic carbon content was determined by drying the sediments, removing inorganic carbon by acid vapor treatment, and analysis on a CE‐440 elemental analyzer from Exeter Analytical (Chelmsford, MA).

To determine a measure of overall pyrethroid toxicity to *H. azteca* at each site while accounting for differences in compound toxicity, sediment pesticide concentrations were converted to toxic units (TUs) based on *H. azteca* 10‐day median lethal concentration (LC_50_) toxicity values for each pesticide measured (Amweg, Weston, & Ureda, 2005; Maund et al., 2002). First, pyrethroid concentrations were normalized to sediment organic carbon (OC). TUs were calculated as TU = [actual pyrethroid sediment concentration (μg/g OC)]/[*H. azteca* 10 day LC_50_ (μg/g OC)](Amweg et al., 2006). TUs were then summed across all pyrethroids measured at a given site, yielding a measure of potential total pyrethroid toxicity at each site (Table S2). Sum TU values were non‐normally distributed (Shapiro–Wilks test; *p*‐value = 1.5 × 10^−5^) but had equal variance (Levene's test; *p*‐value = 1.0 × 10^−1^). Thus, a two‐sided nonparametric Mann–Whitney *U* test was applied using R (v. 3.3.2; R Core Team, 2016) to check for differences between sum TUs at LowPU (*n* = 7) versus HighPU (*n* = 8) sites.

### 2.3 Toxicity testing

The collected *H. azteca* were size‐fractionated and when possible, only juveniles were selected for toxicity testing, defined as those passing through a 600‐μm screen, but retained on a 500‐μm screen. When this size class was not present in sufficient numbers, we broadened the range of sizes used (see Table S3). Body length (rostrum to base of telson) was then measured from a subsample of approximately 30 individuals from each site. Representatives of the same size class used for length measurement and toxicity testing were preserved in ethanol for genetic screening.

Pyrethroid sensitivity was assessed in size‐fractionated animals by determining the LC_50_ for the synthetic pyrethroid, cyfluthrin. Tests were conducted in 80 ml of Milli‐Q purified deionized water to which salts and bromide had been added (Borgmann, 1996; Smith, Lazorchak, Herrin, Brewer‐Swartz, & Thoney, 1997). Ten *H. azteca* individuals were added to each beaker, with three replicate beakers per concentration. A 1‐cm^2^ piece of nylon screen was added to each beaker for a substrate. Cyfluthrin solutions were added in an acetone carrier by creating a solution with the highest concentration needed and then diluting it to obtain the lower concentrations. Acetone concentrations were kept equal in all treatments at <40 μl/L, a concentration found to have no effect in solvent controls. LC_50_ estimates were determined using concentration steps separated by a factor of two. Tests were conducted at 23°C with a 16:8 light:dark photocycle. On the second day of the test, 1 ml of a yeast, cerophyll, trout food suspension was added to each beaker, and a 4‐hr period allowed for feeding before the water was replaced with freshly prepared treatment solution. After 96 hr, the tests were terminated and survivors counted. LC_50_ estimates with 95% confidence intervals were derived by the Spearman–Karber method, using CETIS (Tidepool Scientific Software, McKinleyville, CA).

Water was analyzed for pesticides from one concentration step in the midpoint of the concentration range in a composite of freshly prepared solutions from test initiation and the second day water exchange. The deviation from the nominal concentration was used to adjust test results so that LC_50_ data were reported based on actual rather than nominal concentrations. Actual cyfluthrin concentrations averaged 65% of nominal values (range = 32%–93%). The LC_50_ values in the present study were found to have a non‐normal distribution (Shapiro–Wilks test; *p*‐value = 3.3 × 10^−3^) with unequal variance (Levene's test; *p*‐value = 3.3 × 10^−3^). Thus, the LC_50_ values of LowPU (*n* = 6) versus HighPU (*n* = 9) sites were assessed for differences using a two‐sided nonparametric Mann–Whitney *U* test with R (v. 3.3.2; R Core Team, 2016).

### 2.4 DNA extraction

Individuals preserved in ethanol were first examined under 40× magnification to determine sex. Males were preferentially selected for DNA extraction; however, when there were not enough males for analysis and gravid females were present, effort was taken to dissect and remove embryos to avoid potential contamination from offspring DNA. Genomic DNA was extracted from 10 to 20 individual *H. azteca* from each collection using the Qiagen DNeasy^®^ Blood & Tissue Kit (Qiagen, Germantown, MD). Manufacturer's protocols for DNA extraction from tissue were followed with slight modifications. To fully macerate and homogenize the tissue, each individual was placed in a 2‐ml microcentrifuge tube with 180 μl buffer ATL, 20 μl proteinase K (Qiagen) and one 3.2‐mm stainless steel bead. Tubes were homogenized in the TissueLyser LT (Qiagen) for 10–20 min at a rate of 50 oscillations/min. After maceration, microcentrifuge tubes were incubated overnight (16–24 hr) at 56°C. Genomic DNA was measured for purity (260/280 ratio) and nucleic acid concentration with a spectrophotometer (NanoDrop 2000, Thermo Scientific, Waltham, MA).

### 2.5 Resistance mutation genotyping analysis

Because the current investigation involved genotyping a large number of animals across diverse species groups, a rapid assay was needed to quickly screen for the presence of resistant alleles in the *vgsc*. Based on previous results (Weston et al., 2013), a direct sequencing assay for genotyping the M918 and L925 loci of the Domain II S4‐S6 linker region of the *vgsc* was developed (see Supplemental Methods). In particular, this assay makes use of specific primers that were designed based on their conservation across different *H. azteca* species groups (Table S4). For each collection site from which genotypes could be successfully obtained using the assay, a minimum of 10 individuals were genotyped (with the exception of Owens River) in order to detect resistance mutations at a frequency of 5% or greater within the population. To perform the genotyping assay, a 543‐bp segment (or 578 bp in the UCB population only) of the *vgsc* was amplified in 50‐μl reactions using primer pair VI (Table S4) and the Phusion Hot Spot II High Fidelity Green Taq Polymerase Master Mix (Thermo Fisher Scientific, Waltham, MA), with 5 μl of individual *H. azteca* gDNA. Thermocycler settings were 98°C for 30 s; 35 cycles of 98°C for 10 s, 64.2°C for 30 s, and 72°C for 30 s; and 72°C for 10 min. After bands were confirmed on an agarose gel, they were cleaned with the QIAquick PCR Purification Kit (Qiagen) with a 40‐μl elution volume. Between 200 and 300 ng of cleaned PCR product was sent to the Massachusetts General Hospital DNA Core (Cambridge, MA) for sequencing on an ABI3730XL 96‐capillary DNA Analyzer with internal Rt primer VII (Table S4). Resulting sequence files were examined for quality. If sequence data were of poor quality (i.e., low signal strength or the appearance of a compression), sequencing reactions were repeated using primers IV through VII (in that order, Table S4). Reasons for poor sequence quality likely include poor template fit or the presence of indels in a heterozygous state within the amplicon.

After a sequence of sufficient quality was obtained for each individual, all sequences were trimmed and aligned with CLC Workbench v.7.8 (https://www.qiagenbioinformatics.com/) and manually scored for *vgsc* M918 and L925 loci genotype. Because both alleles were sequenced simultaneously for each individual, homozygotes presented as a singular peak, while heterozygotes presented as two approximately equal peaks at the same locus. Secondary peaks less than 30% of the primary peak height at a locus were not recognized as true heterozygotes, as small secondary peaks can be an indication of baseline noise or contamination (i.e., true contamination or offspring alleles). Most calls were clear, and any ambiguous sequences were discarded and the assay repeated for that individual.

### 2.6 Cytochrome *c* oxidase I genotyping

We assessed species diversity among the populations by analyzing the nucleotide sequence in a 670‐bp region of the mitochondrial cytochrome *c* oxidase I (COI), a region that has proven effective in species identification within North American *Hyalella* (Major, Soucek, Giordano, Wetzel, & Soto‐Adames, 2013; Wellborn & Broughton, 2008; Witt et al., 2006). The target region of COI was PCR‐amplified using the primer pairs I, II, or III (Table S4) for five to 10 individuals per site. PCR was performed in 40‐μl reactions using GoTaq^®^ Green Master Mix (Promega Corporation, Madison, WI) with standard protocols. Cycling conditions were 5 min at 94°C; 40 cycles of 30 s at 94°C, 30 s at 52°C, and 45 s at 72°C; and 5 min at 72°C. PCR products were gel‐purified, and sequenced with the primer IV (Table S4) using an ABI 3730 automated sequencer.

### 2.7 *H. azteca* species determination

Putative species designation was determined primarily using COI sequences from five to 10 *H. azteca* from each collection site. COI sequences were aligned using the Geneious alignment tool implemented in Geneious (version 8.1.3). The amphipod *Parhyale hawaiensis* was included as an outgroup. Relationships among populations were analyzed using the MrBayes (Huelsenbeck & Ronquist, 2001; Ronquist & Huelsenbeck, 2003) tree analysis tool in Geneious. To evaluate species identity, we used NCBI Blast searches to compare our genetically distinct groups (established in the Bayesian analysis) with sequences in the NCBI nucleotide database.

Species group affiliation was inferred for most of the remaining individuals from each collection not included in the COI analysis by utilizing a 327‐bp segment of the *vgsc* generated during the resistance mutation genotyping assay. Each sequence represented a composite of both alleles for a single individual and was manually checked for and marked with IUPAC ambiguity codes at heterozygous loci. Individuals with insufficient sequence quality or length were removed from the analysis, so that the final analysis included 142 individuals (of the 161 genotyped at the *vgsc*). Sequences were aligned using MUSCLE in MEGA v 7.0 (Kumar, Stecher, & Tamura, 2016). After alignment, a maximum‐likelihood (ML) tree was generated using PhyML online (Guindon et al., 2010; http://www.atgc-montpellier.fr/phyml/). The DNA substitution model (HKY85 + G + F, gamma shape parameter = 0.325) was chosen automatically using the Akaike information criterion (AIC=2429.14522). Support values of greater than 90% (1,000 bootstrap replicates) were retained on the unrooted circular cladogram (Figure S1). Branching patterns and clade groupings were largely paralleled in the *vgsc* tree when compared to the COI tree. Thus, despite the selection occurring at the *vgsc*, an evolutionary signal was still present for most species groups within the *vgsc*, with the exception of species E and Ps 28 which were indistinguishable from one another by the *vgsc* tree alone. Putative species designations based on the COI analysis were then overlaid onto the branching patterns of the *vgsc* segment ML tree in order to provide support for species designations for individuals not included in the COI analysis.

For each collection, species designations were necessary to (i) document the species‐level variability at each site and within the overall study, and (ii) determine whether it was reasonable to assume that each collection was indeed a population, or instead a composite of multiple populations (i.e., different species). Final species designations for each genotyped individual were based first on the COI analysis for the subsample of individuals at each site, and then on the corroborating *vgsc* ML analysis if that individual's *vgsc* segment was of sufficient length and quality to be included in the analysis. Finally, if a *vgsc*‐genotyped individual was not included in the *vgsc* ML analysis (as was the case for 19 individuals), then we assumed that the select individual was in the same species group as all of the other individuals in that collection (based on both the COI and *vgsc* analyses), but only if both gene analyses indicated only a single species at that site for all other individuals analyzed from that site.

## 3 RESULTS

### 3.1 Pyrethroids in sediment

We sampled a broad geographic distribution of sites throughout California including eight “low pyrethroid use (LowPU) expected” sites and eight “high pyrethroid use (HighPU) expected” sites (see Table S1). Of the eight LowPU sites, seven contained no detectable pyrethroids in the sediments, and only Bassey Spring Creek contained measurable residues (38.8 ng/g permethrin; Table S2). In contrast, pyrethroids were found in the sediments at all eight HighPU sites, confirming our a priori expectation (Table S2). Bifenthrin was the most common pyrethroid of concern, with additional contributions to total pyrethroids from cyfluthrin, cypermethrin, cyhalothrin, deltamethrin, esfenvalerate, and permethrin.

**Figure 1:**
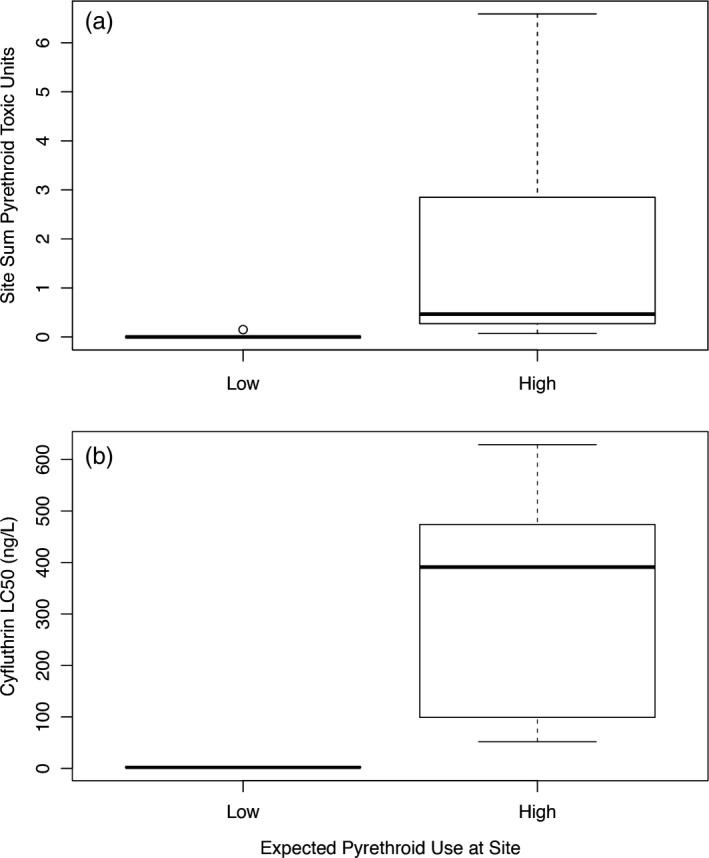
Boxplots illustrating differences in pyrethroid toxic units and *H. azteca* cyfluthrin LC_50_s between expected low pyrethroid use (LowPU) and expected high pyrethroid use (HighPU) sites in California. (a) Pyrethroid sum toxic units (relative to sensitive *H. azteca* toxicity) were greater in HighPU sites (median = 0.47) compared to LowPU collection sites (median = 0; two‐sided Mann–Whitney *U* = 1, *n*_Low_ _PU_ = 7, *n*_HighPU_ = 8, *p *=* *1.5 × 10^−3^). (b) The measured cyfluthrin LC_50_ was higher in *H. azteca* collected from HighPU sites (median = 391 ng/L) compared to LowPU sites (2.1 ng/L; two‐sided Mann–Whitney *U* = 0, *n*_LowPU_ = 6, *n*_HighPU_ = 9, *p *=* *4.0 × 10^−4^)

To determine a measure of pyrethroid sediment toxicity to wild‐type *H. azteca* at each site, pesticide measurements were converted to toxic units (TUs). TUs were generated by normalizing pesticide concentrations for sediment organic carbon content and then dividing them by 10‐day *H. azteca* sediment LC_50_s (Amweg et al., 2005, 2006; Maund et al., 2002). For reference, in this instance, a TU value of 1 indicates that mortality of 50% of wild‐type *H. azteca* individuals would be expected over a 10‐day exposure period at the observed pyrethroid concentration. As the pyrethroids all share a common mode of action and have demonstrated additivity (Trimble, Weston, Belden, & Lydy, 2009), TU values were summed for each site, and values ranged from 0 to 0.15 in LowPU sites and from 0.07 to 6.59 in HighPU sites (Table S2). Summed TUs significantly differed between the two pyrethroid exposure groups, with HighPU sites having higher sediment TU values than LowPU sites (two‐sided Mann–Whitney *U* = 1, *n*_LowPU_ = 7, *n*_HighPU_ = 8, *p *=* *1.5 × 10^−3^; Figure 1).

### 3.2 *H. azteca* species determination

**Figure 2:**
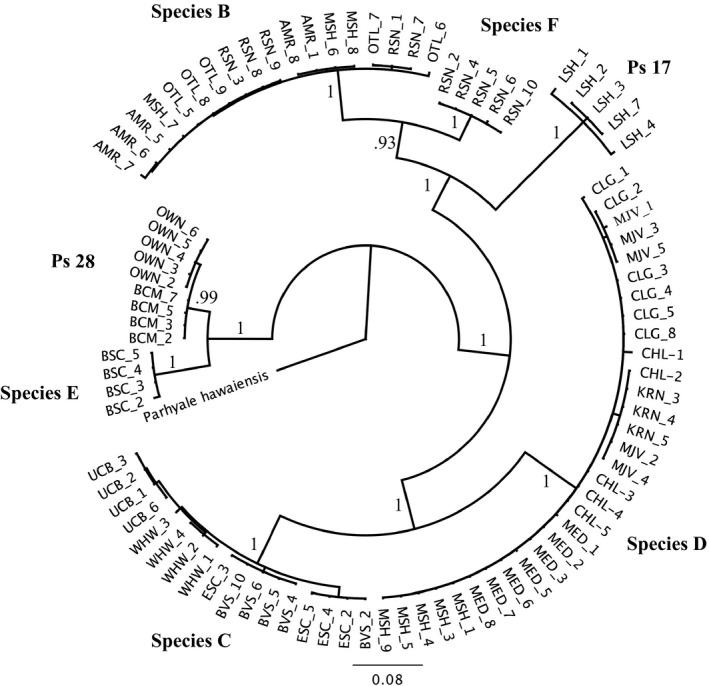
Phylogenetic relationships established with cytochrome *c* oxidase I (COI) sequences among *H. azteca* amphipods from 16 sample sites in California, and one laboratory culture (UCB). The analysis revealed seven well‐supported phylogenetic groups, which we interpret as species. Branch values indicate posterior probabilities from the Bayesian analysis. For site abbreviations, see Table1

Based on our phylogenetic analysis of populations at 16 sites, we identified seven well‐supported, phylogenetically distinct groups (Figure 2), which we interpret as seven separate species. Divergence in pairwise mitochondrial COI sequences among these groups ranged between 10% and 23%, and in most comparisons were near or greater than 20% (Table S5). Previous studies of diversity in the *H. azteca* species complex have demonstrated reproductive isolation among phylogenetic groups with similar levels of COI divergence (Wellborn, Cothran, & Bartholf, 2005; Witt & Hebert, 2000). Five of the seven putative species groups in our study have been reported previously, while the other two appear to be new species. Groups labeled as species B, C, or D (Figure 2) were found in an earlier study of pyrethroid resistance in California (Weston et al., 2013). Moreover, species C has a very broad distribution across much of the United States (Major et al., 2013; Wellborn & Broughton, 2008). The species “Ps 17” and “Ps 28” were previously reported in the Great Basin of California and Nevada (Witt et al., 2006). Two phylogenetic groups, species E and species F, were not similar to COI sequences in the National Center for Biotechnology Information (NCBI; https://www.ncbi.nlm.nih.gov) database and represent new putative species. Each of these new species occurred at only one location (Table 1).

**Table 1:** Study collection sites for *H. azteca*, species designations, cyfluthrin toxicity, and wild‐type (wt) and resistant (res) amino acid frequencies at two voltage‐gated sodium channel (Vgsc) loci associated with pyrethroid resistance

Collection	Site code	Median* Cyfluthrin 96‐hr LC50 (ng/L)	Species^a^	Sample size (*n*)	Vgsc amino acid frequencies				
					M918		L925		
					M (wt)	L (res)	L (wt)	I (res)	V (res)
Laboratory animals									
University of California Berkeley Laboratory Population	UCB	4.7*	C	10	1.00	0	1.00	0	0
Low pyrethroid use (LowPU) expected									
Bassey Spring Creek	BSC	3.8	E	na	na	na	na	na	na
Little Shasta River	LSH	2.1*	Ps 17	10	1.00	—	1.00	—	—
Outlet Creek	OTL	na	B	10	1.00	—	1.00	—	—
Burcham Creek	BCM	na	Ps 28	na	na	na	na	na	na
Owens River	OWN	2.9	Ps 28	1	1.00^b^	—	—	1.00^b^	—
South Fork Kern River	KRN	na	D	10	1.00	—	1.00	—	—
Mojave River	MJV	1.7*	D	10	1.00	—	1.00	—	—
Russian River	RSN	na	B	8	1.00	—	1.00	—	—
			F	2	1.00^b^	—	1.00^b^	—	—
High pyrethroid use (HighPU) expected									
American River	AMR	72*	B	20	1.00	—	0.20	0.80	—
Mosher Slough	MSH	99	B	8	1.00	—	0.06	0.94	—
			D	12	1.00	—	—	0.87	0.13
Chualar Creek	CHL	>492	D	10	0.15	0.85	0.95	0.05	—
Calleguas Creek	CLG	456	D	10	1.00	—	—	1.00	—
Medea Creek	MED	552*	D	10	1.00	—	—	1.00	—
Whitewater River	WHW	na	C	10	1.00	—	—	1.00	—
Buena Vista Creek	BVS	391	C	10	1.00	—	—	1.00	—
Escondido Creek	ESC	189	C	10	1.00	—	—	1.00	—

Summary of data collected from *H. azteca* sp. sourced from waterways in the state of California between October 2014 and August 2015 (see Table S1 for collection details). When possible, cyfluthrin toxicity was assessed. If replicate cyfluthrin tests were performed, LC_50_ values are reported as medians and denoted by an asterisk (*); otherwise, LC_50_ values are indicative of single test measurements (see Table S3). Select parameters were not assessed (“na”). For some collections, sample sizes were insufficient to assess cyfluthrin toxicity, and thus, no LC_50_ value is available for collections from OTL, BCM, KRN, RSN, and WHW. Sample number (*n*) refers to the number of individuals successfully analyzed for genotype at loci M918 and L925 in the Vgsc, and all frequencies were observed (not estimated). Collections from BSC and BCM could not be successfully assessed for *vgsc* genotype and are not reported. For amino acid frequencies, “‐” is equivalent to a frequency of zero.

a Species designation was made based on combined cytochromes oxidase I (COI) genotype data and voltage‐gated sodium channel (*vgsc*) gene fragment sequence data in each population. In cases where two species were identified at the same collection site, amino acid frequencies are provided for each species (RSN and MSH).

b Allele frequencies for populations with fewer than five individuals genotyped should be regarded with caution because of low sample size. Only one individual from OWN (Ps 28) and two individuals from RSN (species F) were successfully genotyped, so reported allele frequency is only a reflection of those individuals.

For each site, five to 10 individuals were sequenced at COI for species determination. We used the 327‐bp segment of the *vgsc* from the pyrethroid resistance genotyping assay as a nuclear marker to make species determinations for the additional individuals in each population (see Supplemental Results and “Suppl_file2_vgsc_sequences_FASTA.txt”). Although the *vgsc* is a marker under selection, our maximum‐likelihood analysis showed that sequences at these loci were divergent enough to signal evolution at the species level rather than evolution of resistance alleles, as strongly supported clades within the analysis paralleled COI‐derived clades, regardless of resistance genotype. Species group was only inferred by the *vgsc* when the lineages were supported (>90%) by COI and *vgsc* data, and thus only for individuals falling into species C, D, or Ps17 (Figure S1).

### 3.3 Pyrethroid sensitivity

Four populations from LowPU sites were highly sensitive to pyrethroids when tested in the laboratory. Cyfluthrin sensitivities in these populations were in the range of 1.7–3.8 ng/L (96‐hr LC_50_; Table 1). Although one LowPU site, Bassey Spring Creek, had measurable permethrin in its sediment, concentrations were below those likely to cause mortality in *H. azteca* (Amweg et al., 2005), and the cyfluthrin 96‐hr LC_50_ recorded for this population (3.8 ng/L) was comparable to the other LowPU populations. For comparison, UCB *H. azteca* representing the US Lab Strain, a well‐characterized wild‐type baseline population (Major et al., 2013), had a median cyfluthrin LC_50_ of 4.7 ng/L and range of 4.3–4.9 ng/L in individual tests (Table S3). In previous tests with this culture, estimates have ranged from 1.7 to 4.3 ng/L (Weston & Jackson, 2009; Weston et al., 2013). Thus, in the absence of appreciable pyrethroid exposure, cyfluthrin LC_50_s of wild populations were consistently <5 ng/L, and consistent with animals from the standard laboratory populations widely used for toxicity testing throughout the United States.

In contrast to cyfluthrin sensitivities determined at the LowPU sites, all toxicity tests of seven *H. azteca* populations from HighPU sites demonstrated that these populations were up to 325‐fold more pyrethroid‐tolerant (Table 1). *H. azteca* populations from HighPU sites exhibited LC_50_s at least one, and typically two, orders of magnitude greater (72–552 ng/L) than those recorded in populations from LowPU sites or the laboratory strain. Toxicity tests with *H. azteca* from HighPU sites yielded significantly higher LC_50_ values than those from LowPU sites with median LC_50_ values of 391 and 2.1 ng/L, respectively (two‐sided Mann–Whitney *U* value = 0, *n*_LowPU_ = 6, *n*_HighPU_ = 9, *p *=* *4.0 × 10^−4^; Figure 1).

Cyfluthrin sensitivity was best explained by expected pyrethroid use on the surrounding land and not by other variables such as species classification or body size. We found that species affiliation alone was not a good predictor of cyfluthrin sensitivity. Nonresistant populations (including the laboratory population) belonged to one of five species (Ps 17, Ps 28, B, C, or D), and resistant *H. azteca* consisted of either species B, C, or D (Table 1). Mean body length of animals tested ranged from 2.3 to 5.3 mm depending on the animals available for testing at each site, but size did not appear to exert a major influence on pesticide sensitivity (Table S3). Two size classes (defined by sieve mesh sizes) from the American River population were tested, and their LC_50_s varied by approximately a factor of two, comparable to variation in multiple tests within a given size class (e.g., LC_50_s for Mosher Slough, Chualar, and Medea Creeks). This variation in LC_50_s between size classes was small compared to variation in pesticide resistance between populations.

### 3.4 Pyrethroid resistance alleles

**Figure 3:**
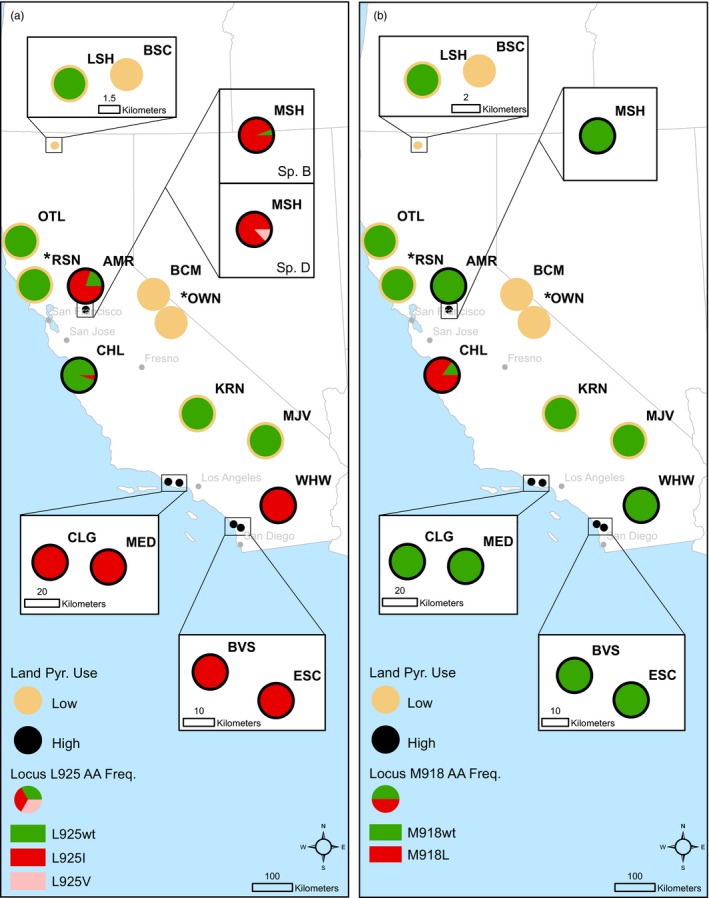
Map of *H. azteca* sampling sites, expected site pyrethroid use classification, and allele frequencies for the Vgsc amino acid (AA) substitutions associated with pyrethroid resistance. Tan sites are expected to have low pyrethroid use (LowPU); black sites are expected to have high pyrethroid use (HighPU). Red or pink portions of pie charts indicate resistance alleles; green portions of pie charts indicate wild‐type alleles. Allele frequencies were only presented for populations from which data are available for five individuals or more. An asterisk (*) at RSN and OWN designate at least one population at each site for which allele frequencies were not presented because of low sample size. When more than one species was designated at a site and allele frequencies were different by species, species are displayed separately. (a) Allele frequencies at Vgsc locus 925. (b) Allele frequencies at Vgsc locus 918

At all seven sites with demonstrated tolerance (defined as 10‐fold or higher cyfluthrin LC_50_ compared to wild type), resistance allele frequencies were 80% or higher for one of three Vgsc amino acid substitutions associated with pyrethroid resistance: M918L, L925I, or L925V (Table 1; Figure 3). In contrast, only wild‐type alleles were identified at LowPU sites, with the exception of the L925I mutation that was present in the only individual that was successfully genotyped from Owens River.

A total of 161 *H. azteca* were successfully genotyped at both the M918 and L925 loci of the voltage‐gated sodium channel. These two loci are sites of nonsynonymous base pair substitutions previously identified in resistant *H. azteca* (Weston et al., 2013). The genotyping assay developed in the present study was successful for genotyping most populations of *H. azteca*. However, for two of the most divergent lineages of *H. azteca* within this study (species E and Ps 28), the genotyping assay was unsuccessful in discerning *vgsc* genotypes at the M918 and L925 loci. The populations for which this limitation was reached include individuals from Bassey Spring Creek (species E), Burcham Creek, and Owens River (both Ps 28), although a single individual was successfully genotyped from Owen's River (Table 1). The *vgsc* primers used during the present study were ineffective in amplifying the region of interest in these groups, likely due to sequence divergence. However, they were successfully used to amplify the *vgsc* segment and determine allele frequencies across organisms from the 13 remaining field sites (five LowPU sites, eight HighPU sites) and the UCB laboratory population.

**Figure 4:**
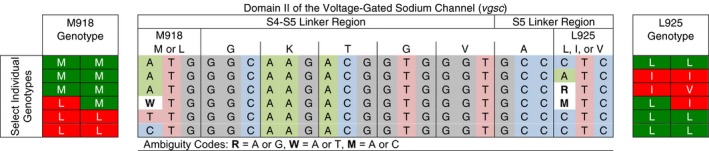
Example variation of individual genotypes at loci M918 and L925 in the Vgsc from *H. azteca* in the present study. Ambiguity codes are used to denote heterozygous allele states in example individuals because both alleles were sequenced simultaneously

The most common resistance allele identified in the *H. azteca* populations from HighPU sites was the Vgsc L925I amino acid substitution, originally linked to pyrethroid resistance in the pest whitefly, *Bemisia tabaci* (Alon et al., 2006), and previously identified in pyrethroid‐tolerant populations of *H. azteca* species B and D from California (Weston et al., 2013). Of the 100 genotyped individuals from HighPU sites, 87 harbored at least one L925I allele, and 79 were homozygous for that resistance allele (Table S6). The L925I resistance allele frequencies ranged from 0.80 to 1.00 for most populations (Figure 3a). In Chualar Creek, the only HighPU site where the L925I allele frequency was low (0.05), the population instead had a high frequency of the M918L mutation (see below; Table 1; Figure 3b). One individual even harbored both the M918L and L925I pyrethroid resistance alleles, each in a heterozygous state (Figure 4).

At the M918 locus, all individuals from the UCB laboratory population and most individuals from field collections (151 out of 161) were homozygous wild type for methionine (Figure 3b). Resistance mutations at M918 were only identified in the 10 individuals from the Chualar Creek (species D) population. Among the individuals genotyped from this HighPU site, three different alleles were identified at the M918 locus (Table S6). While both of the alleles M and L_CTG_ have been previously documented in *H. azteca* (Weston et al., 2013), the L_TTG_ allele is a newly documented resistance mutation variant.

A variant at the Vgsc L925 locus, a leucine‐to‐valine (L925V) amino acid substitution was identified in some *H. azteca* from Mosher Slough (HighPU). In *H. azteca*, the L925V allele was only identified in species D organisms, although both species B and D organisms were collected from Mosher Slough. In fact, of the species D animals collected from Mosher Slough, none harbored the L925 wild‐type allele; all genotypes consisted of only resistance alleles (L925I or L925V, Table 1; Figure 3). The species B individuals collected from Mosher Slough also had a high frequency of the L925I resistance allele, but there was a single L925 wild‐type allele in the eight individuals genotyped (Table 1; Figure 3a).

## 4 DISCUSSION

### 4.1 Pyrethroid resistance in *H. azteca* is correlated with pyrethroid exposure

Resistance is both widely distributed across agricultural and urban areas of California and geographically predictable. Our a priori, largely land use‐based classifications regarding pyrethroid use successfully predicted the *H. azteca* populations that exhibited resistance phenotypes and genotypes, mainly falling within the southern and western portions of the state (Figure 3). Sediment analysis confirmed the presence of measurable pyrethroids at all HighPU sites, with only one LowPU site registering any measurable pyrethroids, at levels too low to cause toxicity in *H. azteca*. Most of the state's major population centers lie within this region, as well as the most intensively farmed lands. At least 470,000 kg of pyrethroids are used annually in California (CDPR, 2015), and retail sales, which are not included in the available data, would only add to this total. This amount represents a tripling of use of the compounds since 1990. The amount used is approximately equally divided between agricultural applications and urban/residential applications for structural pest control and landscape maintenance.

These uses appear responsible for pyrethroid‐driven evolution in *H. azteca*, a finding supported by numerous reports of pyrethroid‐related sediment (Amweg et al., 2006; Holmes et al., 2008; Phillips et al., 2012; Weston et al., 2005) and water (Weston & Lydy, 2010, 2012; Weston et al., 2009) toxicity to wild‐type *H. azteca* throughout California. In particular, seasonal rain and irrigation patterns in California have been shown to facilitate the movement of pyrethroids into aquatic environments. Lethal quantities of pyrethroids introduced during episodic rain events (Weston & Lydy, 2010, 2012; Weston et al., 2009) would cause sensitive *H. azteca* to perish, leaving only those with mutations that confer resistance (i.e., M918L, L925I, and L925V) to repopulate the water body.

### 4.2 Decreased pyrethroid sensitivity is explained by the presence of resistance alleles at high frequencies

Despite the genetic variation present between members of the *H. azteca* species complex, the increased tolerance to cyfluthrin observed in HighPU populations is best explained by the presence of resistance alleles. Although others have demonstrated differences in chemical sensitivity among members of the *H. azteca* species complex (Leung, Witt, Norwood, & Dixon, 2016; Soucek, Mount, Dickinson, Hockett, & McEwen, 2015), we found no relationship between species group affiliation and pyrethroid sensitivity in the present study. The lack of correlation is best illustrated by the dramatic differences in sensitivities among species C and D organisms. For example, species C individuals in pyrethroid‐affected waterways in southern California (Buena Vista and Escondido Creeks) were 40–83 times more resistant to cyfluthrin than the UCB laboratory species C. Similarly, compared to nonresistant species D at the LowPU Mojave River site, the species D populations collected from HighPU sites (Calleguas and Medea Creeks) were approximately 300 times more resistant to cyfluthrin. In fact, the largest disparity in sensitivities between any two populations in the present study existed between two species D populations.

Only one *H. azteca* collection site at which cyfluthrin sensitivity was determined, Mosher Slough, contained both species B and D organisms (40% and 60% of the individuals at the site, respectively). Thus, the resulting cyfluthrin LC_50_ is a measure of sensitivity across a mixture of two species groups rather than a true measure of population sensitivity. The tolerance recorded in organisms collected from Mosher Slough was still well above (21‐ to 45‐fold) the sensitive laboratory *H. azteca*, suggesting that both species B and D in this population were displaying a resistance phenotype. In addition, both species B and D populations from Mosher Slough had allele frequencies above 0.94 for resistance alleles, further linking pyrethroid resistance alleles to resistance phenotypes.

In addition to identifying the same L925I and M918L resistance mutations we previously identified in *H. azteca* (Weston et al., 2013), we also found two novel resistance alleles in *H. azteca*: the M918L conferred by a TTG codon rather than CTG and the L925V amino acid substitution. The M918L_TTG_ and the M918L_CTG_ were both identified in the Chualar Creek (HighPU) population, and have also been identified in populations of the green peach aphid, *M. persicae* (Panini et al., 2015). The leucine‐to‐valine (L925V) amino acid substitution was identified in some *H. azteca* from Mosher Slough (HighPU). Novel in *H. azteca*, this nonsynonymous base pair substitution has been associated with pyrethroid resistance in *Varroa destructor*, a parasitic mite of the Western honey bee *Apis mellifera* (Gonzalez‐Cabrera, Davies, Field, Kennedy, & Williamson, 2013). In *H. azteca*, the L925V allele was only identified in species D organisms, although both species B and D organisms were collected from Mosher Slough.

The presence of resistance alleles at all HighPU sites and their absence from all LowPU sites (with the exception of one individual from Owen's River) provides strong support that decreased pyrethroid sensitivity is conferred via pyrethroid exposure‐driven selection for resistance alleles. However, our resistance allele genotyping assay reached its limit among two of the most divergent lineages of *H. azteca* (species E and Ps 28), leaving the allele frequencies within these species groups uninvestigated. The singular resistant L925I homozygous genotype scored for the only individual sequenced from Owens River, a LowPU site, merits further investigation. Nevertheless, frequencies of the L925I mutation in HighPU populations of *H. azteca* (typically 0.8–1) were higher than allele frequencies observed in the pest whitefly, where this amino acid substitution was first identified. For example, in cypermethrin‐resistant populations of *B. tabaci* (Q‐biotype) from China collected in 2010, the L925I resistance mutation frequencies ranged from 0.40 to 0.70 (Yuan et al., 2012). The M918L mutation frequencies in the Chualar Ceek *H. azteca* population were much higher (L_TTG_ = 0.70 and L_CTG_ = 0.15) than the same mutation frequencies (less than 0.5) found in populations of the green peach aphid, *Myzus persicae* documented by Panini et al. (2015). It is possible that the Chualar Creek *H. azteca* in our study had higher frequencies of M918L because it is functionally more favored in *H. azteca* than in *M. persicae*. Panini et al. (2015) attributed the low frequencies of the M918L mutation in the aphid to the high cost of this mutation compared with more prevalent resistance mutations near the M918L site (e.g., M918T and L1014F) (Panini et al., 2015). Alternatively, this high frequency in Chualar Creek *H. azteca* may be attributed to reduced opportunity for gene flow in *H. azteca* (Wellborn & Capps, 2012) compared to *M. persicae*.

In some populations of *H. azteca*, the L925I allele appears to be fixed in the population. *H. azteca* populations from HighPU sites, specifically in species C (Whitewater Creek, Buena Vista Creek, and Escondido Creek) and species D (Calleguas Creek, Medea Creek) individuals, showed no evidence of wild‐type alleles. However, our sample sizes were small (10 individuals for each collection site), making a true designation of L925I fixation in these populations tenuous. An analysis with more individuals for each population would be needed to discern fixation more confidently. Nevertheless, the absence of a wild‐type allele in five populations across two species groups suggests sufficient selective pressure to drive high frequencies of the L925I allele. Further, because some of the apparently L925I‐fixed are species C, they can provide a novel study system to better understand the dynamics and consequences of pyrethroid selective pressures in this model organism. In our previous work, we designated the US Lab strain (Major et al., 2013) commonly used as a model ecotoxicology organism in the United States, as species C. This species has been identified in several field collections across the United States including in Florida (Major et al., 2013) and Oklahoma (Wellborn & Broughton, 2008). The existence of species C animals in the present study near the California–Mexico border (at Whitewater River, Buena Vista, and Escondido Creeks) is novel in the state of California. Further, the increased pyrethroid tolerance and the fixed L925I resistance allele within these populations offer a unique opportunity for directly comparing the sensitive US Laboratory *H. azteca* species C with resistant wild populations of the same species.

The L925I resistance allele was identified at high frequencies across three different species: B, C, and D. In addition, a novel mutation in *H. azteca*, L925V, was identified in one population of species D, and two different M918L alleles (codon TTG or CTG) were identified in another species D population. Taken together, these resistance mutations indicate at least six independent evolutionary events produced widespread resistance in the *H. azteca* species complex throughout California. Given that the sampling sites were deliberately selected to preclude *H. azteca* movement through direct hydrological connections, the very limited opportunity for gene flow suggests the possibility that resistance evolved independently at the various sites, making the emergence of resistance even more frequent than the six occasions we can definitively document.

Unlike the well‐documented resistance in pests specifically targeted by pyrethroid application, resistance in *H. azteca* is particularly remarkable given that it is an aquatic invertebrate. There are no approved aquatic uses of pyrethroids in California; they are used exclusively in terrestrial habitats. If the compounds remained on the lands to which they were applied, *H. azteca* and other aquatic organisms would have no exposure to pyrethroids. Yet the frequencies of M918L and L925I mutations in populations of pyrethroid‐resistant *H. azteca* exceed the documented frequencies of the same alleles evolved in parallel in some pyrethroid‐resistant pest insects (Panini et al., 2015; Yuan et al., 2012). The mutant alleles appear fixed in the populations at five of the eight HighPU sites we investigated. It follows that selection imposed by pyrethroids, entering aquatic systems indirectly and inadvertently, is acting on populations of *H. azteca* with comparable or greater evolutionary pressure than that applied to pest insects via direct, targeted application. The fact that resistance is geographically widespread and appears in multiple species within the *H. azteca* complex provides stark evidence that current approaches to mitigate off‐site movement of pesticide residues are grossly inadequate.

### 4.3 Evolution of pyrethroid resistance is constrained in *H. azteca*

The high prevalence of the L925I mutation across three different species groups and the presence of only three resistance mutations found in all populations combined suggest that evolution is constrained in *H. azteca*. The L925I mutation may be preferred because the subtle change from leucine to isoleucine at this locus has been demonstrated to effectively prevent pyrethroid binding without grossly changing the shape of the protein (O'Reilly et al., 2006). Although we only obtained a 327‐bp sequence fragment in the present study, we previously showed that either L925I or M918L alone is sufficient to provide resistance (Weston et al., 2013), and therefore, each allele investigated in the present study likely harbors a single resistance mutation. If these populations follow the same pattern observed previously in other California *H. azteca* populations, as lacking additional mutations on the *vgsc* (Weston et al., 2013), they are only utilizing 5% of the 41 possible amino acid positions documented by Dong et al. (2014) to provide pyrethroid resistance in different arthropod species. However, even if we take the most conservative view and only consider the area sequenced, we find substitutions in only two of the eight possible amino acid positions shown previously to confer resistance in insects.

Pyrethroid resistance in other insect pests has also been shown to be restricted to only a few mutations. In the tobacco budworm, *Heliothis virescens*, only three mutations have been recorded (Dong et al., 2014). A close investigation of the most widely observed mutations, V410M and L1014H, found that the L1014H was more effective at increasing pyrethroid tolerance and had a limited impact on the normal functioning of the Vgsc. The authors also noted that L1014H is replacing the V410M mutation in natural populations, likely because of the increased efficiency and decreased costs associated with the mutation (Zhao, Park, & Adams, 2000). Overall, it appears that not all possible mutations provide a similar level of net benefits across species when considering both protection against pyrethroids versus detrimental impacts to protein function.

Protein evolution is constrained to particular genes and specific sites due to trade‐offs between the net benefit of the mutation and antagonistic pleiotropy, or the negative costs of the mutations on the natural structure and function of the enzyme (Stern, 2013). With at least 121 documented cases of pyrethroid resistance caused by mutations in the *vgsc* across 55 species (Dong et al., 2014), the *vgsc* may be considered a “hot spot” for molecular evolution (although Feyereisen et al. (2015) reviewed alternative resistance mechanisms). Martin and Orgogozo (2013) argue that this is not surprising due to the specificity of insecticide resistance mutations. For example, by affecting the binding site for a pesticide or toxin, they provide the desired trait (e.g., resistance) while affecting few other physiological processes. Therefore, there have been many examples of parallel evolution in studies of insecticide resistance. Fewer studies consider the differences in the specific amino acid substitutions across species or why only a few of the possible mutations are selected by a particular organism. Storz (2016) argues that genetic background plays a role in further constraining molecular evolution, which would explain both why resistance to pyrethroid pesticides tends to converge on the *vgsc* and why certain taxa such as *H. azteca* have “preferred” amino acid substitutions.

### 4.4 Pyrethroid resistance in *H. azteca* has ecological and evolutionary implications

In the present study, existence of pyrethroid resistance alleles explained the decreased sensitivity observed in *H. azteca* from all sites between Sacramento and San Diego, a distance of nearly 800 km. Not only are pyrethroid resistance alleles common and widespread in populations of *H. azteca* from areas of anticipated pyrethroid use in California, but their multiple origins indicate they are a common, repeated solution to pyrethroid selective pressure in the environment within members of this species complex.

The ecological and evolutionary implications for the strong selective pressures provided by pyrethroids are numerous. For populations of *H. azteca* that are sensitive to pyrethroids but lack standing genetic diversity for resistant genotypes, exposure to pyrethroids could lead to population collapse. For populations with sufficient size and standing genetic variation, evolutionary rescue may occur (Bell & Gonzalez, 2009), but could be associated with “genetic erosion” or a reduction in genetic diversity through a bottleneck and subsequent founder effect (Van Straalen & Timmermans, 2002). In support of this theory, there is evidence that pyrethroid‐resistant H. azteca exhibit increased fitness costs, including a decrease in thermal tolerance and a tendency for greater sensitivity to other chemicals (Heim et al., 2018). In addition, given their ability to survive at concentrations of pyrethroids two orders of magnitude higher than wild‐type animals, increased bioaccumulation of pyrethroids in resistant *H. azteca* has been shown, leading to an increased risk of pyrethroid trophic transfer to their fish predators (Muggelberg et al., 2017).

Pyrethroid use and reported environmental effects occur on a global scale (Li, Cheng, Wei, Lydy, & You, 2017), and there is no biological reason to presume the selective pressures and resulting genetic changes we documented are restricted to California. Pyrethroids in surface waters and associated toxicity have been reported in many regions of the United States (Hintzen, Lydy, & Belden, 2009; Kuivila et al., 2012; Rogers et al., 2016). Internationally, they have been found in water bodies at concentrations of ecotoxicological concern in several South American countries (Hunt et al., 2016), England (Long, House, Parker, & Rae, 1998), Spain (Feo, Ginebreda, Eljarrat, & Barceló, 2010), China (Mehler, Li, Lydy, & You, 2011), and Australia (Jeppe et al., 2017). Additionally, while most studies documenting aquatic risk of pyrethroids in the United States have generally relied upon *H. azteca*, work elsewhere has reported toxicity to *Chironomus dilutus* (Mehler et al., 2011) and the amphipod, *Austrochiltonia subtenuis* (Jeppe et al., 2017). Thus, it is possible, and even likely, that other species are exposed to the same pyrethroid‐related selective pressures we documented in *H. azteca*. The genetic consequences of exposure to pyrethroids specifically, and pesticides in general, may be pervasive but largely overlooked by the traditional environmental monitoring tools of bioassessments and toxicity testing. Therefore, there is a need for the increased application of genetic tools in environmental assessment to fully comprehend the extent of evolutionary impact of anthropogenic contaminants.

## DATA ARCHIVING STATEMENT

Data used in these analyses and primer sequences used for sequencing as well as a FASTA file demonstrating *vgsc* sequence variation from the genotyping assay are provided in the Supplemental Information. COI sequences for the two newly identified species, species E and F, were deposited in the NCBI GenBank sequence databank (accession numbers MG488280–MG488288).
