# Supplementary material for: Unintentional exposure to terrestrial pesticides drives widespread and predictable evolution of resistance in freshwater crustaceans
Source: Evol Appl. 2018 Jan 20;11(5):748–61. doi: 10.1111/eva.12584 (PMC5979619; doi:10.1111/eva.12584)
Supplement: Supplementary file 1 [file EVA-11-748-s001.docx]

**SUPPORTING INFORMATION**

Unintentional exposure to terrestrial pesticides drives widespread and predictable evolution of resistance in freshwater crustaceans

Kaley M. Major^a^, Donald P. Weston^b^, Michael J. Lydy^c^, Gary A. Wellborn^d^, Helen C. Poynton^a,*^

^a^ School for the Environment, University of Massachusetts, Boston MA

^b^ Department of Integrative Biology, University of California, Berkeley CA

^c^ Center for Fisheries, Aquaculture and Aquatic Sciences, and Department of Zoology, Southern Illinois University, Carbondale, IL

^d^ Department of Biology, University of Oklahoma, Norman, OK

*Correspondence and requests for materials should be addressed to H.C.P. (email:helen.poynton@umb.edu)

**SUPPLEMENTAL METHODS**

**Site selection, sampling, and classification**

Most sites were in separate watersheds either flowing to the Pacific Ocean, the salinity of which *H. azteca* cannot tolerate, or in an endorheic basin with no outlet. There were only a few exceptions to the genetic isolation of the sites. The American River and Mosher Slough sites are both within the San Francisco Bay watershed, and the Kern River site used to be, but the river’s flow has not reached the Bay in many decades due to agricultural diversions. Also, water from the Bassey Spring Creek site flows to the Little Shasta River site during winter months.

All sites were visited between October 2014 and August 2015, and *H. azteca* were collected using a D-framed net. After collection at most sites, organisms were transported to the laboratory with continuous aeration and used toxicity testing, typically two days after collection. The animals were size fractionated to obtain a suitable testing group, with the specific size range chosen depending upon the individuals available. A subsample of this group was preserved in 100% ethanol for genetic sequencing. However, at some sites densities were too low to permit toxicity testing (<100 individuals collected), and in those cases, individuals were preserved in ethanol on site.

Since there is no direct application of pyrethroid insecticides to aquatic systems, exposure of *Hyalella azteca* and other aquatic invertebrates is most likely to come from runoff from treated agricultural lands, runoff from urban and residential areas where pyrethroids are used for landscape application and structural pest control, and discharge of municipal wastewater. Prior to any analysis of material collected during the present study, the sampling locations were grouped into two categories depending upon the anticipated likelihood of pyrethroid use on surrounding land, given the known sources. The categories were: “low pyrethroid use (LowPU) expected”, and “high pyrethroid use (HighPU) expected”. Assignment was based on the following considerations:

1. Identification of surrounding land uses using satellite photography;
2. Reconnaissance of nearby land uses during sampling; and,
3. Past sampling of the waterbodies and reports of pyrethroid-related toxicity to laboratory-cultured, wild-type *H. azteca.*

The rationale for our *a priori* placement of each site into its designated category is described below.

**Low pyrethroid use (LowPU) expected**

Bassey Spring Creek – Bassey Spring emerges from the ground approximately 1 km upstream of the sampling site, and forms Bassey Spring Creek. Before reaching the sampling site it passes only a couple homes and a few farm buildings. Land along the creek is irrigated pasture used for cattle grazing, and use of insecticides on pasture is extremely rare. Beyond the immediate valley in which the creek lies, the area is undeveloped grasslands and forest.

Little Shasta River – Headwaters of the river lie in undeveloped forestlands. At lower elevations, the river passes through irrigated pasture, beginning 2 km upstream of the sampling site, and continuing downstream. Much of this land is grazed year-around by cattle, though some is periodically cut for hay. Both uses would rarely require insecticides. There is some production of alfalfa, which could require an annual pyrethroid application, but alfalfa fields are few in number, and are sprinkler irrigated (rather than flood irrigated) which would lessen offsite movement of pesticides (S. Orloff, pers. comm.). In the entire county (Siskiyou County) on all crops there are only 287 kg of pyrethroids used annually (2015 data; CDPR, 2015), compared to tens of thousands of kilograms used in the counties in the High PU category.

Outlet Creek- The surrounding area is heavily forested. Agriculture is limited to a few isolated fields for grazing, none close to the site. The nearest significant residential development is in the city of Willits (population approximately 5,000), 13 km distant.

Russian River – The primary land use along the Russian River in the vicinity of the sampling site is production of wine grapes. There is little pyrethroid use on this crop. Only 4.5 kg of pyrethroids are used annually on wine grapes in the entire county in which the site lies (Sonoma County; 2015 data(CDPR, 2015)). The potential for pyrethroid inputs from the scattered cities and towns along the river was also considered, but viewed as not significant. The nearest city is Healdsburg, California, 4 km upstream of the sampling site. Stormwater input to the river would be minimized by the relatively small size of the community (approximately 12,000 residents). Municipal wastewater flow averages six million liters per day, is tertiary treated, and discharged to a percolation pond that seeps into the river, minimizing the potential for pyrethroids reaching the river.

Burcham Creek – The site is in a sparsely-populated, arid region along the California-Nevada border. Agriculture is limited to livestock grazing. The nearest community is Walker, California, with a population of 700 residents, located 13 km downstream from the sampling site.

Owens River – The sampling site is at the headwaters of the Owens River, on the forested eastern flanks of the Sierra Nevada Mountains. The site is remote from any population center of appreciable size; the agriculture along the riverbanks is limited to scattered fields used for livestock grazing.

South Fork Kern River – The surrounding lands are mountainous, forested, and partially within the Sequoia National Forest. Irrigated pastureland is present along the riverbanks, but this agricultural use would not typically require pyrethroid application. The population is limited to scattered small towns, the nearest being Onyx, California with a population of approximately 500. Approximately 100 km to the west, the river flows out of the mountains and enters the Central Valley of California, an area of intensive agriculture with heavy pyrethroid use. However, between the sampling site and the agricultural lands is a dammed reservoir and a 730 m drop in elevation as the river quickly descends to the valley floor through a steep canyon. Thus the potential for *Hyalella* gene flow from the agricultural valley to the mountainous sampling area is negligible.

Mojave River – This site is in the Mojave Desert. Except during rare flood events, the Mojave River flows underground through porous strata. It only emerges from the ground at three locations along its entire length, and it remains on the surface only for a short distance before again disappearing below ground. At the time of sampling, the river was visible on the ground surface at the sampling location for <0.1 km. Agriculture is non-existent, and the nearest community of appreciable size (Barstow, California) is 70 km upstream.

**High pyrethroid use (HighPU) expected**

American River – Several studies have documented that the portion of the river flowing through the major urban area of Sacramento, California and surrounding communities receives stormwater inputs of bifenthrin and other pyrethroids during winter rains (Weston, Chen, et al., 2015; Weston and Lydy, 2010, 2012; Weston, Schlenk, et al., 2015).

Mosher Slough – This site is in the densely populated city of Stockton, California. The slough receives urban runoff, but it also passes through areas of intensive agriculture prior to entering the city. It has previously been shown to have pyrethroids in the sediment (Weston et al., 2013).

Chualar Creek – This waterbody is a tributary of the Salinas River, which flows through a heavily farmed valley producing crops commonly treated with pyrethroids (e.g., lettuce). Sediment in the Salinas River and its tributaries in this agricultural area have been shown to be toxic to *H. azteca*, with pyrethroids likely responsible (Anderson et al., 2003; Anderson et al., 2006; Ng et al., 2008).

Calleguas Creek – There are many potential pyrethroid sources in the area. The lands immediately surrounding the sampling site are intensively farmed for a wide variety of crops (e.g., strawberries, cucumbers), and farther upstream the creek carries runoff from much of Camarillo, California (population approximately 67,000). In addition, the Camarillo municipal wastewater treatment plant discharges to a tributary (Conejo Creek) 7 km upstream of the sampling site. Previous work has shown widespread toxicity throughout the watershed to a variety of test species, including *H. azteca* (Anderson et al., 2002). Toxicity was attributed to organophosphate insecticides, however this work was done before awareness of pyrethroid pesticide toxicity, and these compounds were not included among the analytes.

Medea Creek – The sampling location is on the outskirts of Los Angeles, on the downstream border of Agoura Hills, California, and with the city of Oak Park farther upstream. Both Agoura Hills and Oak Park are largely residential (combined population approximately 35,000), and urban runoff from these properties flows into Medea Creek. Farther downstream from the sampling site, the creek joins other tributaries that drain additional urban lands.

Whitewater River – The site is on the northern edge of California’s Salton Sea, in the Coachella Valley, known for its agricultural production (e.g., dates, citrus crops, grapes, bell peppers). During the dry season, water in the river consists of irrigation return flow from agriculture and municipal wastewater treatment plant effluent. During periods of heavy rain in the winter, the cities of Palm Springs, Indio, Coachella, and others discharge urban stormwater to the river.

Buena Vista Creek – Much of the upper portion of the creek’s course lies within the greater San Diego metropolitan area. The creek flows from Vista to Carlsbad, California, a distance of 14 km, all of which is densely populated, and the sampling site is located within this reach.

Escondido Creek – The creek’s watershed is heavily urbanized. As it passes through Escondido, California (population approximately 150,000), the creek is a concrete-lined stormwater channel that carries urban runoff. The sampling site is on the downstream edge of Escondido after the creek returns to its natural bed, but water quality at the site would clearly be influenced by inputs to the upstream channelized portion.

**Pesticide sediment analyses**

Sediment samples were extracted using a matrix-dispersive accelerated solvent extraction method (You et al., 2008) using a Dionex 200 with 33 ml stainless steel cells with 1:1 dichloromethane: acetone (v/v) at 100 ºC and 1500 pound-force per square inch (psi) for two 5 min static cycles. Extracts were collected in 60 ml glass collection vials (Dionex, Sunnyvale, CA, USA), solvent exchanged to hexane, concentrated to 1 ml, and eluted through a dual layer solid phase extraction cartridge (SPE) containing 300 mg of graphitized black carbon, 600 mg of primary/secondary amine and capped with Na_2_SO_4_. The SPE was primed with 3 ml of hexane prior to the introduction of the extract. The target pesticides were eluted with 10 ml of 1:1 hexane:acetone (v/v) solution. The eluents were solvent exchanged to 0.1 % acetic acid in hexane with a final volume of 1 ml and the acidification step was used to avoid isomerization of the pyrethroids (You and Lydy, 2007).

Final extracts were analyzed using an Agilent 6850 gas chromatograph 5975 XL mass spectrometer (GC-MS; Agilent Technologies, Palo Alto, CA). A 2 µl sample was injected into the gas chromatograph using pulsed splitless mode. The oven was set at 50 °C for 1 min, heated to 200 °C at 20 °C/min, then to 295 °C and held at 205 °C for 5 min. Quantification was performed using internal standard calibration. Calibration curves were based on area using concentrations of 2, 5, 10, 50, 100, 250, 500 ng/ml of each pesticide and surrogate, while the concentrations of the internal standards were kept constant at 20 ng/ml for each standard.

**Resistance mutation genotyping assay development**

A direct sequencing assay for genotyping the M918 and L925 loci of the Domain II S4-S6 linker region of the voltage gated sodium channel (*vgsc*) was developed because select mutations at these sites were previously correlated with *H. azteca* cyfluthrin resistance( Weston et al., 2013). To design and validate the genotyping assay, previously-designed primers (Weston et al., 2013) (**Table S4**) were used to PCR-amplify and clone the desired region of interest from 1-5 individuals from a subset of the populations in the present study: University of California Berkeley (UCB) laboratory, Calleguas Creek (CLG), American River (AMR), and Mosher Slough (MSH). The *H. azteca* from these collections were previously documented to include at least three putative species groups(Weston et al., 2013). Identifying conserved regions of the *vgsc* across multiple *H. azteca* sp. ensured that the genotyping assay would be robust for multiple species of the *H. azteca* complex. PCR reactions (20 µl) contained:10 µl of Brilliant III Ultra-Fast SYBR® Green QPCR Master Mix with Low ROX (Agilent Technologies, Santa Clara, CA), 1 µl of 10 µM primer pair I (**Table S4**), 4 µl nuclease free water and 5 µl gDNA. Thermocycler PCR settings were 95 °C for 5 min; 35 cycles of 95 °C for 30 s, 58.5 °C for 30 s, and 68 °C for 45 s; 68 °C for 15 min. Successfully-amplified PCR products were cloned using the pCR®4-TOPO® TA Cloning® Kit with One Shot® TOP10 Chemically Competent *E. coli* (Invitrogen, Carlsbad, CA). To ensure that both alleles along this stretch of the *vgsc* were scored for each *H. azteca*, five clones per individual were picked, checked for the desired amplicon, grown in LB broth, purified with the QIAprep Spin Miniprep Kit (Qiagen) and sequenced with plasmid primers T7 and T3 (**Table S4**) on an ABI3730XL 96-capillary DNA Analyzer (Applied Biosystems, Foster City, CA) at the MGH DNA Core Facility (Cambridge, MA).

Once sequences for all clones were visualized in CLC Workbench v 7.8 (https://www.qiagenbioinformatics.com/) and aligned using MEGA v.7.0.20(Kumar et al., 2016), alleles at the M918 and L925 loci were recorded for each individual, and a new collection of Rt primers were designed to regions that were highly conserved in all four cloned populations. These new primers (**Table S4**) were created to target a smaller amplicon of the *vgsc* for the genotyping assay and/or act as internal sequencing primers to reduce the possibility of sequencing nontarget amplification products. The first step in executing the direct sequencing resistance mutation genotyping assay was the amplification of a 543 bp segment (or 578 bp in the UCB population only) of the *vgsc* using primer pair VI (**Table S4**) for the same individuals for which cloned genotypes were available. PCR reaction volumes (50 µl) each contained: 25 µl Phusion Hot Spot II High Fidelity Green Taq Polymerase Master Mix (ThermoFisher Scientific, Waltham, MA), 17.5 µl nuclease free water, 2.5 µl of 10 µM primer pair II, and 5 µl of individual *H. azteca* gDNA. Thermocycler settings were 98 °C for 30 s; 35 cycles of 98 °C for 10 s, 64.2 °C for 30 s, and 72 °C for 30 s; 72 °C for 10 min. After bands were confirmed on an agarose gel, they were cleaned with the QIAquick PCR Purification Kit (Qiagen) with a 40 µl elution volume. Cleaned PCR products were analyzed for DNA concentration using a NanoDrop 2000 (Thermo Scientific, Waltham, MA). Between 200 and 300 ng of cleaned PCR product was sent to the MGH DNA Core (Cambridge, MA) for sequencing on an ABI3730XL 96-capillary DNA Analyzer with internal Rt primer VII (**Table S4)**. Sequences were aligned and visualized in CLC Workbench v 7.8 to manually call M918 and L925 genotypes. All 10 of the individual genotypes scored by cloning were consistent with the results of the genotyping assay, thereby providing assay validation.

**SUPPLEMENTAL RESULTS**

**Inferring species-level determination using a nuclear marker**

A nuclear gene marker, a 327-bp segment of the voltage-gated sodium channel (*vgsc*) Domain II S4-S6 region, was used to infer species identification as a secondary method when COI data were not available for an individual. The *vgsc* segment maximum likelihood (ML) analysis yielded highly supported (>90%) branches on the *vgsc* tree that corresponded to species level distinctions for putative species C, D, and Ps 17 (**Figure S1**). Species B and species F formed a single monophyletic group based on *vgsc* sequences alone, and therefore the *vgsc* sequence analysis was not informative for inferring species B from species F. Based on the COI analysis of the subsample of each site collection (and later supported by the *vgsc* ML analysis), most sites (14) harbored a single species of *H. azteca*. Only two collections showed the presence of two species at the same site: Russian River and Mosher Slough. In the case of Russian River, all individuals were identified to species through the COI analysis given that this collection was composed of species B and species F, which are otherwise indistinguishable using *vgsc* sequence data. For the Mosher Slough collection, both species B and D were identified in the COI analysis subsample, so a total of 20 individuals were genotyped for the *vgsc* and included in the *vgsc* ML analysis. The resulting *vgsc* gene tree offered high support (>95%) in resolving these two species, so all individuals from Mosher Slough were categorized as either species B or species D by a combination of COI data for (six individuals) and *vgsc* segment data (20 individuals).

|  |
| --- |

Figure S1. Unrooted maximum likelihood (ML) cladogram of *H. azteca* based on a 327-bp segment of the voltage gated sodium channel (*vgsc*). Terminal node labels represent individuals (see Table 1 for abbreviation details), and bootstrap branch supports (1000 replicates) above 90 are displayed. Outer circle labels represent putative species distinctions made by cytochrome *c* oxidase I (COI) analysis of a subset of *H. azteca* individuals within the *vgsc* cladogram. Based on the *vgsc* analysis, species C, D, and Ps 17 form distinct groups. Putative species B and F form a separate clade from other species groups, but are indistinguishable from one another based on the *vgsc* tree.

Table S1. California sampling sites, including the downstream waterbodies, latitude/longitude, and the land uses in the vicinity of the sampling sites.

|  | Site Name | | Watershed | Location  (Latitude, Longitude) | Land Use^a^ | Sampling Date |
| --- | --- | --- | --- | --- | --- | --- |
|  | |  |  |  |  |  |
| **Low pyrethroid use (LowPU) expected** | | | | | | |
|  | Bassey Spring Creek (BSC) | | Klamath River to Pacific Ocean | 41.7172 , -122.3471 | F, R | June 2015 |
|  | Little Shasta River (LSH) | | Klamath River to Pacific Ocean | 41.7114 , -122.3833 | F, R, C^b^ | June 2015 |
|  | Outlet Creek (OTL) | | Eel River to Pacific Ocean | 39.5319, -123.4050 | F | June 2015 |
|  | Burcham Creek (BCM) | | Walker River to endorheic basin | 38.3840 , -119.4295 | F, R | July 2015 |
|  | Owens River (OWN) | | Owens River to endorheic basin | 37.7499, -118.9379 | F | July 2015 |
|  | South Fork Kern River (KRN) | | Kern River to endorheic basin | 35.6908, -118.2348 | F, R | May 2015 |
|  | Mojave River (MJV) | | Mojave River to endorheic basin | 35.0380 , -116.3814 | D | May 2015 |
|  | Russian River (RSN) | | Russian River to Pacific Ocean | 38.5664 , -122.8515 | U, C | Aug. 2015 |
|  |  | |  |  |  |  |
| **High pyrethroid use (HighPU) expected** | | | | | | |
|  | American River (AMR) | | Sacramento River to San Francisco Bay | 38.5658 , -121.3838 | U | Nov. 2014 |
|  | Mosher Slough (MSH) | | San Joaquin River to San Francisco Bay | 38.0325, -121.3654 | U, C | Oct. 2015 |
|  | Chualar Creek (CHL) | | Salinas River to Pacific Ocean | 36.5583, -121.5296 | C | Oct. 2014 |
|  | Calleguas Creek (CLG) | | Calleguas Creek to Pacific Ocean | 34.1644, -119.0612 | U, C | July 2015 |
|  | Medea Creek (MED) | | Malibu Creek to Pacific Ocean | 34.1401, -118.7600 | U | June 2015 |
|  | Whitewater River (WHW) | | Whitewater River to endorheic basin | 33.5694, -116.1080 | U, C | May 2015 |
|  | Buena Vista Creek (BVS) | | Buena Vista Creek to Pacific Ocean | 33.1814, -117.3218 | U | June 2015 |
|  | Escondido Creek (ESC) | | Escondido Creek to Pacific Ocean | 33.0986, -117.1306 | U | June 2015 |
|  | |  |  |  |  |  |

^a^F = Forests; R = Rangeland; D = Desert; U = Urban; C = Cropland

^b^Nearby croplands are largely irrigated pasture, on which insecticides are rarely used.

Table S2. Total organic carbon (%) and pyrethroid concentrations (ng/g) followed by pesticide toxic units (TU)^a^ in brackets, measured in surficial sediments at the collection sites.

|  |  |  | Pyrethroid | | | | | | |  | |
| --- | --- | --- | --- | --- | --- | --- | --- | --- | --- | --- | --- |
|  | Site Code | Org. C | Bifenthrin | Cyfluthrin | Cypermethrin | Cyhalothrin | Permethrin | Deltamethrin | Esfenvalerate | Site Sum TU | |
| **Low pyrethroid use (LowPU) expected** | | | | | | | | | | |  |
|  | BSC | 2.42 | nd | nd | nd | nd | 38.8 [0.15]^b^ | nd | nd | 0.15 | |
|  | LSH | 6.82 | nd | nd | nd | nd | nd | nd | nd | 0.00 | |
|  | BCM | 16.50 | nd | nd | nd | nd | nd | nd | nd | 0.00 | |
|  | OWN | 2.29 | nd | nd | nd | nd | nd | nd | nd | 0.00 | |
|  | KRN | 4.41 | nd | nd | nd | nd | nd | nd | nd | 0.00 | |
|  | MJV | 0.64 | nd | nd | nd | nd | nd | nd | nd | 0.00 | |
|  | RSN | 1.48 | nd | nd | nd | nd | nd | nd | nd | 0.00 | |
|  |  |  |  |  |  |  |  |  |  |  | |
| **High pyrethroid use (HighPU) expected** | | | | | | | | | | |  |
|  | AMR | 3.26 | 1.2 [0.07] | nd | nd | nd | nd | nd | nd | 0.07 | |
|  | MSH**^c^** | 1.70 | 50.0 [5.66] | 2.4 [0.13] | 3.7 [0.60] | 1.4 [0.18] | 9.2 [0.05] | nd | nd | 6.59 | |
|  | CHL | 2.06 | 27.8 [2.60] | 3.0 [0.13] | nd | 3.3 [0.36] | 158.5 [0.71] | nd | 3.0 [0.09] | 3.89 | |
|  | CLG | 2.96 | 2.9 [0.19] | nd | nd | nd | nd | nd | nd | 0.19 | |
|  | MED | 3.90 | 30.2 [1.49] | 2.5 [0.06] | 2.0 [0.14] | 1.5 [0.09] | 15.4 [0.04] | 1.2 [0.04] | nd | 1.81 | |
|  | WHW | 2.75 | 3.1 [0.22] | nd | nd | nd | 4.3 [0.01] | nd | 3.5 [0.08] | 0.35 | |
|  | BVS | 1.68 | 2.9 [0.33] | nd | nd | nd | nd | nd | nd | 0.36 | |
|  | ESC | 0.77 | 2.3 [0.57] | nd | nd | nd | nd | nd | nd | 0.57 | |

No sediment collected at Outlet Creek (OTL). “Nd” indicates not detected (<1 ng/g).

**^a^**TU for each site was calculated as the measured concentration divided by the *Hyalella azteca* 10-d sediment toxicity values for each pesticide: bifenthrin=0.52 **μ**g/g OC, cyfluthrin=1.08 **μ**g/g OC, cypermethrin=0.38 **μ**g/g OC, cyhalothrin=0.45 **μ**g/g OC, permethrin=10.83 **μ**g/g OC, deltamethrin = 0.79 **μ**g/g OC, esfenvalerate= 1.54 **μ**g/g OC) as determined elsewhere (Amweg et al., 2005; Maund et al., 2002).

**^b^**Bassey Spring Creek was the only LowPU site that yielded measurable pyrethroids. The source of permethrin at this site is presumed to be landscape use at one of the few home or other structures that lie between where the spring emerges from the ground and the sampling site.

^c^Mosher Slough data from June 2015, four months before *H. azteca* collection (unpublished data; S. Nutile, Southern Illinois University).

Table S3. 96-h LC_50_ values for cyfluthrin toxicity tests with the University of California Berkeley (UCB) laboratory population and 11 field-collected populations of *H. azteca*.

|  | **Site Code** | **Screens^a^**  **(µm)** | **Body length**  **(mm; mean** ± s.d.**)** | **Control survival (%)** | **LC50**  **(ng/L (95% CI))** |
| --- | --- | --- | --- | --- | --- |
|  | | | | | |
| **Laboratory Animals** | | | | | |
|  | UCB laboratory | 500-600  500-600  500-600  500-600 | no data  no data  no data  no data | 100  97  100  98 | 4.3(3.4-5.5)  4.9(4.0-5.9)  4.4(3.5-5.5)  4.9(3.9-6.0) |
| **Low pyrethroid use (LowPU) expected** | | | | | |
|  | | | | | |
|  | BSC | 1000-2000 | 5.3±0.5 | 85 | 3.8(3.2-4.7) |
|  |  |  |  |  |  |
|  | LSH | 600-1000  600-1000 | 3.3±0.6  3.3±0.6 | 93  90 | 2.0(1.6-2.4)  2.2(1.8-2.8) |
|  |  |  |  |  |  |
|  | OWN | 1000-2000 | 5.0±0.8 | 71 | 2.9(2.3-3.9) |
|  |  |  |  |  |  |
|  | MJV | 500-2000  500-2000 | 4.2±1.4  4.2±1.4 | 74  75 | 1.8(1.3-2.4)  1.5(1.1-2.0) |
|  |  |  |  |  |  |
| **High pyrethroid use (HighPU) expected** | | | | | |
|  | | | | | |
|  | AMR | 500-600  600-1000 | 2.3±0.3  3.0±0.4 | 78  93 | 52(42-65)  92(67-125) |
|  |  |  |  |  |  |
|  | MSH | 500-600  500-600  500-600 | 2.4±0.3  no data  no data | 75  90  85 | 99(80-123)  193(158-226) **^b^**  211(176-244) **^b^** |
|  |  |  |  |  |  |
|  | CHL | 1000-2000  500-600  500-600 | 4.9±0.7  no data  no data | 88  78  80 | >492  535(403-650) **^b^**  353(255-441) **^b^** |
|  |  |  |  |  |  |
|  | CLG | 500-1000 | 3.2±0.8 | 88 | 456(364-569) |
|  |  |  |  |  |  |
|  | MED | 500-1000  500-1000 | 3.3±0.7  3.3±0.7 | 88  78 | 629(504-785)  474(423-532) |
|  |  |  |  |  |  |
|  | BVS | 500-1000 | 3.1±0.5 | 83 | 391(302-506) |
|  |  |  |  |  |  |
|  | ESC | 500-1000 | 2.9±0.7 | 92 | 189(131-272) |

Data from multiple tests with the same population are shown when available. No data are presented for OTL, BCM, KRN, RSN, and WHW because the number of individuals available was insufficient for toxicity testing.

**^a^**Screen sizes are given as the size on which the animals were retained, and the size through which they passed.

**^b^**Data previously reported (Weston et al., 2013).

|  |  |  | Product Size by Population  (bp) | | PCR Annealing Temp  (**°**C) |
| --- | --- | --- | --- | --- | --- |
| Primer/  Primer Pair | Left Primer (5’ – 3’) | Right Primer (5’ – 3’) | UCB | CLG |  |
| COI amplification | | | | | |
| I^a^ | GTTATATAAACTATTAGCCTTCCAA | ACTGCCACAACAGAYAARTAMGACCC | 670 |  | 52.0 |
| II^a^ | GTTATATAAACTATTAGCCTTCCAA | ACAGCAACAACAGATAARTARGACC | 670 |  | 52.0 |
| III^a^ | GTTATATAAACTATTAGCCTTCCAA | ATCAAAATATACACYTCTGGGTGVCC | 670 |  | 52.0 |
| COI sequencing | | | | | |
| IV^a^ | GTTATATAAACTATTAGCCTTCCAA |  |  |  |  |
| *vgsc* amplification (Cloning) | | | | | |
| V^a^ | AGGGTGTTCAAGCTCGCTAA | TGTCGGTGCATGAAACTCAC | 670 | 635 | 58.5 |
| *vgsc* sequencing (Cloning)^b^ | | | | | |
| T7 | TAATACGACTCACTATAGGG |  |  |  |  |
| T3 |  | ATTAACCCTCACTAAAGGGA |  |  |  |
| *vgsc* amplification (genotyping assay) | | | | | |
| VI^a^ | AGGGTGTTCAAGCTCGCTAA | ACATGCTCTCGATCCACTCC | 578 | 543 | 64.2 |
| *vgsc* sequencing (genotyping assay) | | | | | |
| VII |  | GGCCGTCTTGAGACCATTT | 489 | 456 |  |
| VIII |  | ACATGCTCTCGATCCACTCC | 578 | 543 |  |
| IX |  | ACGATCATGAACGAGTGGAA | 538 | 504 |  |
| X^a^ | AGGGTGTTCAAGCTCGCTAA |  | 578 | 543 |  |
| XI |  | AATTCTTGCCGAAGAGTTGC | 154 | 154 |  |

Table S4. Primers used during the cytochrome oxidase I (COI) sequencing and the determination of voltage gated sodium channel (*vgsc*) genotypes at the M918 and L925 loci in the Domain II S4-S6 linker region in *H. azteca*

^a^ Primer pair I-III, primer IV, primer pair V, Lt primer of primer pair VI, and primer X were previously designed (Weston et al., 2013).

^b^ Primers T7, T3 are from Invitrogen pCR®4-TOPO® TA Cloning® Kit (Invitrogen, Carlsbad, CA)

|  | | | | | | |
| --- | --- | --- | --- | --- | --- | --- |
|  | Proportion of differing base pair composition^a^ | | | | | |
|  | Species C | Species D | Ps 17 | Ps 28 | Species E | Species F |
| Species B | 0.18 | 0.20 | 0.20 | 0.22 | 0.23 | 0.10 |
| Species C | - | 0.14 | 0.22 | 0.20 | 0.21 | 0.18 |
| Species D |  | - | 0.23 | 0.21 | 0.20 | 0.18 |
| Ps 17 |  |  | - | 0.21 | 0.22 | 0.19 |
| Ps 28 |  |  |  | - | 0.10 | 0.19 |
| Species E |  |  |  |  | - | 0.21 |
|  |  |  |  |  |  |  |

Table S5. Pairwise genetic dissimilarity between putative *H. azteca* species groups at cytochrome *c* oxidase I (COI).

^a^Within-population dissimilarity values were typically <0.01.

|  |  |  | Locus M918  M (wt); L (res) | | | | | | | Locus L925  L (wt); I (res); V (res) | | | | | | |
| --- | --- | --- | --- | --- | --- | --- | --- | --- | --- | --- | --- | --- | --- | --- | --- | --- |
|  |  |  | Genotype Frequency | | | | Allele Frequency | | | Genotype Frequency | | | | Allele Frequency | | |
| Site Code | *N* | Sp.^a^ | MM  ATG/ATG | ML  ATG/TTG | LL  CTG/CTG | LL  TTG/TTG | M  ATG | L  TTG | L  CTG | LL  CTC/CTC | LI  CTC/ATC | II  ATC/ATC | IV  ATC/GTC | L  CTC | I  ATC | V  GTC |
|  |  |  |  |  |  |  |  |  |  |  |  |  |  |  |  |  |
| UCB | 10 | C | 1.00 | - | - | - | 1.00 | - | - | 1.00 | - | - | - | 1.00 | - | - |
|  |  |  |  |  |  |  |  |  |  |  |  |  |  |  |  |  |
| LSH | 10 | Ps 17 | 1.00 | - | - | - | 1.00 | - | - | 1.00 | - | - | - | 1.00 | - | - |
|  |  |  |  |  |  |  |  |  |  |  |  |  |  |  |  |  |
| OTL | 10 | B | 1.00 | - | - | - | 1.00 | - | - | 1.00 | - | - | - | 1.00 | - | - |
|  |  |  |  |  |  |  |  |  |  |  |  |  |  |  |  |  |
| OWN | 1 | Ps 28 | 1.00 | - | - | - | 1.00 | - | - | - | - | 1.00^b^ | - | - | 1.00^b^ | - |
|  |  |  |  |  |  |  |  |  |  |  |  |  |  |  |  |  |
| KRN | 10 | D | 1.00 | - | - | - | 1.00 | - | - | 1.00 | - | - | - | 1.00 | - | - |
|  |  |  |  |  |  |  |  |  |  |  |  |  |  |  |  |  |
| MJV | 10 | D | 1.00 | - | - | - | 1.00 | - | - | 1.00 | - | - | - | 1.00 | - | - |
|  |  |  |  |  |  |  |  |  |  |  |  |  |  |  |  |  |
| RSN | 8 | B | 1.00 | - | - | - | 1.00 | - | - | 1.00 | - | - | - | 1.00 | - | - |
|  | 2 | F | 1.00 | - | - | - | 1.00^b^ | - | - | 1.00^b^ | - | - | - | 1.00^b^ | - | - |
|  |  |  |  |  |  |  |  |  |  |  |  |  |  |  |  |  |
| AMR | 20 | B | 1.00 | - | - | - | 1.00 | - | - | 0.05 | 0.30 | 0.65 | - | 0.20 | 0.80 | - |
|  |  |  |  |  |  |  |  |  |  |  |  |  |  |  |  |  |
| MSH | 8 | B | 1.00 | - | - | - | 1.00 | - | - | - | 0.13 | 0.87 | - | 0.06 | 0.94 | - |
|  | 12 | D | 1.00 | - | - | - | 1.00 | - | - | - | - | 0.75 | 0.25 | - | 0.87 | 0.13 |
|  |  |  |  |  |  |  |  |  |  |  |  |  |  |  |  |  |
| CHL | 10 | D | - | 0.30 | 0.30 | 0.40 | 0.15 | 0.70 | 0.15 | 0.90 | 0.10 | - | - | 0.95 | 0.05 | - |
|  |  |  |  |  |  |  |  |  |  |  |  |  |  |  |  |  |
| CLG | 10 | D | 1.00 | - | - | - | 1.00 | - | - | - | - | 1.00 | - | - | 1.00 | - |
|  |  |  |  |  |  |  |  |  |  |  |  |  |  |  |  |  |
| MED | 10 | D | 1.00 | - | - | - | 1.00 | - | - | - | - | 1.00 | - | - | 1.00 | - |
|  |  |  |  |  |  |  |  |  |  |  |  |  |  |  |  |  |
| WHW | 10 | C | 1.00 | - | - | - | 1.00 | - | - | - | - | 1.00 | - | - | 1.00 | - |
|  |  |  |  |  |  |  |  |  |  |  |  |  |  |  |  |  |
| BVS | 10 | C | 1.00 | - | - | - | 1.00 | - | - | - | - | 1.00 | - | - | 1.00 | - |
|  |  |  |  |  |  |  |  |  |  |  |  |  |  |  |  |  |
| ESC | 10 | C | 1.00 | - | - | - | 1.00 | - | - | - | - | 1.00 | - | - | 1.00 | - |

Table S6. Genotype (including codon) and allele frequencies for the M918 and L925 Vgsc loci in *Hyalella azteca* from a laboratory population and 14 field sites in California.

^a^ At sites with more than one species (Sp.), genotype and allele frequencies are provided for each putative species.

^b^ Populations with fewer than five individuals genotyped should be regarded with caution because of low sample size. Only one individual from OWN (Ps 28) and two individuals from RSN (species F) were successfully genotyped.

**Literature Cited**

Amweg, E. L., D. P. Weston, and N. M. Ureda. (2005). Use and toxicity of pyrethroid pesticides in the Central Valley California, USA. *Environ. Toxicol. Chem., 24*(4), 966-972.

Anderson, B. S., V. de Vlaming, K. Larsen, L. S. Deanovic, S. Birosik, D. J. Smith, J. W. Hunt, et al. (2002). Causes of ambient toxicity in the Calleguas Creek watershed of Southern California. *Environ. Monit. Assess., 78*, 131-151.

Anderson, B. S., J. W. Hunt, B. M. Phillips, P. A. Nicely, K. D. Gilbert, V. de Vlaming, V. Connor, et al. (2003). Ecotoxicologic impacts of agricultural drain water in the Salinas River, California, USA. *Environ. Toxicol. Chem., 22*(10), 2375-2384.

Anderson, B. S., B. M. Phillips, J. W. Hunt, V. Connor, N. Richard, and R. S. Tjeerdema. (2006). Identifying primary stressors impacting macroinvertebrates in the Salinas River (California, USA): relative effects of pesticides and suspended particles. *Environ. Pollut., 141*(3), 402-408. doi:10.1016/j.envpol.2005.08.056

CDPR. (2015). Pesticide Use Reporting - 2015 Summary Data. Retrieved from http://www.cdpr.ca.gov/docs/pur/purmain.htm

Kumar, S., G. Stecher, and K. Tamura. (2016). MEGA7: Molecular Evolutionary Genetics Analysis Version 7.0 for bigger datasets. *Mol. Biol. Evol., 33*(7), 1870-1874. doi:10.1093/molbev/msw054

Maund, S. J., M. J. Hamer, M. C. G. Lane, E. Farrelly, J. H. Rapley, U. M. Goggin, and W. E. Gentle. (2002). Partitioning, bioavailability, and toxicity of the pyrethroid insecticide cypermethrin in sediments. *Environ. Toxicol. Chem., 21*(1), 9-15.

Ng, C. M., D. P. Weston, J. You, and M. J. Lydy. (2008). Patterns of pyrethroid contamination and toxicity in agricultural and urban stream segments. In J. Gan, F. Spurlock, P. Hendley, & D. Weston (Eds.), *American Chemical Society Symposium Series 991* (pp. 355-369). Washington, D. C.: American Chemical Society.

Weston, D. P., D. Chen, and M. J. Lydy. (2015). Stormwater-related transport of the insecticides bifenthrin, fipronil, imidacloprid, and chlorpyrifos into a tidal wetland, San Francisco Bay, California. *Sci. Total Environ., 527-528*, 18-25. doi:10.1016/j.scitotenv.2015.04.095

Weston, D. P., and M. J. Lydy. (2010). Urban and agricultural soures of pyrethroid insecticides to the Sacramento-San Joaquin Delta of California. *Environ. Sci. Technol., 44*, 1833-1840.

Weston, D. P., and M. J. Lydy. (2012). Stormwater input of pyrethroid insecticides to an urban river. *Environ. Toxicol. Chem., 31*(7), 1579-1586. doi:10.1002/etc.1847

Weston, D. P., H. C. Poynton, G. A. Wellborn, M. J. Lydy, B. J. Blalock, M. S. Sepulveda, and J. K. Colbourne. (2013). Multiple origins of pyrethroid insecticide resistance across the species complex of a nontarget aquatic crustacean, *Hyalella azteca.* *Proc. Natl. Acad. Sci. U.S.A., 110*(41), 16532-16537. doi:10.1073/pnas.1302023110

Weston, D. P., D. Schlenk, N. Riar, M. J. Lydy, and M. L. Brooks. (2015). Effects of pyrethroid insecticides in urban runoff on Chinook salmon, steelhead trout, and their invertebrate prey. *Environ. Toxicol. Chem., 34*(3), 649-657. doi:10.1002/etc.2850

You, J., and M. J. Lydy. (2007). A solution for isomerization of pyrethroid insecticides in gas chromatography. *J. Chromatogr. A., 1166*(1-2), 181-190. doi:10.1016/j.chroma.2007.08.014

You, J., D. P. Weston, and M. J. Lydy. (2008). Quantification of pyrethroid insecticides at sub-ppb levels in sediment using matrix-dispersive accelerated solvent extraction with tandem SPE cleanup. In J. Gan, F. Spurlock, P. Hendley, & D. P. Weston (Eds.), *Synthetic Pyrethroids: Occurrence and Behvaior in Aquatic Environment* (pp. 87-113). New York, NY, USA: Oxford University Press.
